# “Moonlighting” at the power plant: how mitochondria facilitate serial killing by CD8^+^ cytotoxic T cells

**DOI:** 10.1038/s41392-021-00846-3

**Published:** 2021-12-22

**Authors:** Dimitrios Mougiakakos, Sascha Kahlfuss

**Affiliations:** 1 Department of Hematology, Oncology, and Stem Cell Transplantation, Otto-von-Guericke University Magdeburg, Magdeburg, Germany; 2 Institute of Molecular and Clinical Immunology, Medical Faculty, Otto-von-Guericke University Magdeburg, Magdeburg, Germany; 3 Institute of Medical Microbiology and Hospital Hygiene, Medical Faculty, Otto-von-Guericke University Magdeburg, Magdeburg, Germany; 4 Health Campus Immunology, Infectiology and Inflammation (GC-I3), Medical Faculty, Otto-von-Guericke University, Magdeburg, Germany; 5 Center for Health and Medical Prevention (CHaMP), Otto-von-Guericke University, Magdeburg, Germany

**Keywords:** Tumour immunology, Molecular medicine, Translational immunology, Lymphocytes

Griffiths and colleagues recently demonstrated in *Science*^1^ that mitochondrial translation is required by CD8^+^ cytotoxic T cells (CTLs) to facilitate serial killing of target cells.

CD8^+^ CTLs provide immunity to viruses and tumors by acting as serial killers that eliminate infected or transformed target cells one after another. To achieve that, CTLs utilize a cytotoxic protein machinery that involves perforin and granzymes to activate effector caspases and to induce apoptosis in target cells.

T-cell development, activation, and effector function critically rely on intact mitochondria and mitochondrial metabolism. Amongst other functions, mitochondria provide ATP and calcium-buffering for migration and proliferation, they dictate T cells’ differentiation program, and mitochondrial dynamics (i.e., fission and fusion) are central during memory formation. In fact, mitochondria are also important for CTL antitumor immunity, although many of the mechanistic intricacies behind this observation remained so far largely elusive.

Lisci et al. now report that it might be mitochondrial translation, which is required for sustained killing capacity of CTLs.^1^ In one of their previous studies, the authors in a joint effort with other groups and using a novel high-density murine immunophenotyping platform had identified the Ubiquitin carboxyl-terminal hydrolase 30 (USP-30) as a crucial regulator for CTL killing.^2^ Localized in the outer mitochondrial membrane, USP-30 prevents ubiquitination of mitochondria and mitophagy. In their current study, the authors extended on this finding in that they demonstrate that *Usp30*^*−/−*^ CTLs show a disorganized (or completely absent) mitochondrial cristae structure, reduced expression of the mitochondrial translocase of outer membrane 20 (TOM20) and the mitochondrial matrix enzyme pyruvate dehydrogenase, and increased mitophagy. Abolished mitochondrial ultrastructure in *Usp30*^*−/−*^ CTLs correlated with impaired oxidative phosphorylation (OXPHOS). Vice versa, *Usp30*^*−/−*^ CTLs showed increased basal glycolysis but were defective in serial killing.

To test whether defective killing of *Usp30*^*−/−*^ CTLs was a result of reduced OXPHOS, the authors used specific inhibitors of the electron transport chain (ETC) complexes or disrupted the mitochondrial membrane potential. However, none of these experimental interventions impaired the serial killing capacity of CTLs. In addition, even the genetic deletion of NDUFS4, which results in ETC complex I disruption, did not interfere with the ability of CTLs to perform sustained killing. Furthermore, ATP levels in *Usp30*^*−/−*^ CTLs appeared normal, and *Usp30*^*−/−*^ CTLs showed an unaffected motility, had no defect in TCR-induced calcium flux, and could still release (albeit smaller) cytotoxic granules.

**Fig. 1:**
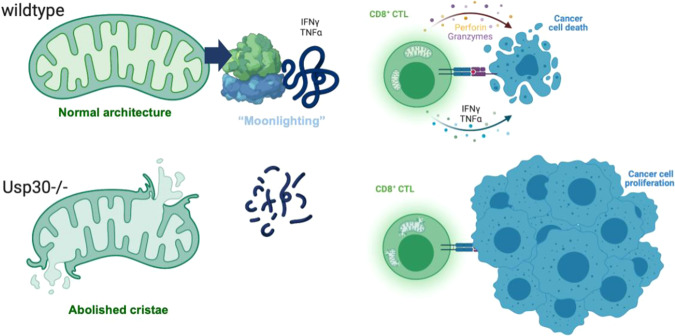
Sustained killing by CD8^+^ CTLs needs mitochondrial translation. During serial killing of malignant target cells (wild type) CD8^+^ CTLs have a high-energy demand to meet, amongst other processes, their increased protein synthesis (i.e., proteome remodeling). Mitochondrial translation and so-called RNA-binding “moonlight” proteins support cytosolic translation of cytolytic proteins (i.e., Perforin and Granzymes) and tumoricidal cytokines including IFN-γ and TNF-α (upper panel). Deletion of USP-30 in CD8^+^ CTLs impacted mitochondrial ultrastructure and mitochondrial translation that elicited the selective mitigation of cytosolic translation of cytolytic proteins and of tumoricidal cytokines cumulating in tumor cell proliferation and cancer progression (lower panel)

Next, the authors measured protein expression levels of granzyme B and perforin. Here, *Usp30*^*−/−*^ CTLs showed reduced granzyme B expression and a maturation defect of perforin. However, impaired protein expression was not due to defective transcription, as *Gzmb* and *Prf1* gene expression in *Usp30*^*−/−*^ CTLs appeared normal. Instead, the authors identified defective mRNA translation in *Usp30*^*−/−*^ CTLs to be causative for reduced granzyme B and perforin protein expression (Fig. 1). In line with this, wild type (WT) CTLs in the presence of the cytosolic protein synthesis inhibitor cycloheximide showed impaired cytotoxicity mirroring the phenotype of *Usp30*^*−/−*^ CTLs.

To elucidate, which class of proteins was affected by the defect in translation in *Usp30*^*−/−*^ CTLs, the authors performed mass spectrometry. Interestingly, in addition to mitochondrial proteins (>70%) that were most likely degraded by mitophagy, granzyme B, and the transcription factor eomesodermin that regulates cytotoxic proteins such as perforin, granzyme B, and interferon-γ (IFN-γ), showed reduced expression in *Usp30*^*−/−*^ CTLs suggesting a rather selective process. In addition, also the expression of TNF-α, IFN-γ, and TNF-β appeared to be impaired in *Usp30*^*−/−*^ CTLs.

In view of the emerging evidence in the field that mitochondrial translation can regulate the effector function of CD8^+^ T cells and CD4^+^ T helper (Th) cells including Th17 cells^3,4^ and as the authors had found mitochondrial and only selected cytosolic proteins to be reduced in *Usp30*^*−/−*^ CTLs, they now tested the hypothesis that direct inhibition of mitochondrial translation is sufficient to mitigate CTL function. When using doxycycline to specifically target mitochondrial protein translation, the authors indeed detected reduced synthesis of granzyme B and perforin that are translated by cytosolic ribosomes and observed impaired killing by WT CTLs.

Together, the data by Griffiths and colleagues^1^ provide novel evidence that mitochondrial translation regulates cytosolic protein synthesis in CTLs to ensure serial killing. In their study, the authors suggest that mitochondrial proteins could act as RNA-binding proteins for specific mRNAs (“Moonlighting”) and thereby mediate cytosolic synthesis of proteins such as granzyme B, TNF-α, and IFN-γ during serial killing of CTLs. In this way, the bioenergetic competence of CTLs is carefully linked to the availability of effector molecules for immune responses that represent energetically demanding situations.

The fact that mitochondrial translation can regulate T-cell function is increasingly recognized in the field and the current study adds compelling evidence for this fascinating mechanism. A (future) comprehensive analysis of the RNA interactome of mitochondrial proteins in CTLs would further complement the present study.

From a clinical perspective, the finding of this study has important implications. First, drugs that interfere with mitochondrial translation such as tigecycline should be used with caution and only when necessary. Second, targeted therapies stabilizing mitochondrial translation in CTLs could amplify serial killing during antitumor or antiviral immune responses. Such an approach could be particularly interesting in the context of emerging adaptive cell therapies using genetically modified immune cells such as chimeric antigen receptor T cells. Appropriate strategies could mean cultivating the immune cells under higher temperatures.^3^ Moreover, ectopic expression of micro RNAs (miRs) such as miR-1 that directly promote mitochondrial translation^5^ seems also conceivable. Whether these therapeutic strategies are actually feasible and effective in amplifying antitumor CTL activity need further investigations and must be proven in controlled clinical trials.
